# Synthetic biological toggle circuits that respond within seconds and teach us new biology

**DOI:** 10.1093/synbio/ysab027

**Published:** 2021-08-28

**Authors:** Sonja Billerbeck

**Affiliations:** Molecular Microbiology, Groningen Biomolecular Sciences and Biotechnology Institute, University of Groningen, Groningen, The Netherlands

Imagine it would take several minutes or even hours for your light bulb to turn on after you hit the switch—not very useful for many daily (and nightly) activities.

Light switches are made from the so-called toggle switches; basic and widely used electrical components that provide binary on–off control over electrical circuits, allowing quick decision-making and memory. Synthetic biologists have built analogues genetic toggle switches but until now those only responded in time-ranges of minutes to hours, limiting their use in applications that require real-time action. Mishra *et al.* have recently built a biological bistable toggle switch in yeast that responds within seconds by mimicking nature’s way of rapid response generation (1).

Synthetic biologists envision to control cellular behavior by engineering biology in analogy to electrical circuits. Implementing a synthetic biological toggle switch was thus one of the early achievements of the field (2). While, over the last two decades, synthetic biologists have mastered to build toggle switches that respond to various inputs—chemicals, light or temperature—and show high switching robustness (3); one challenge remained: timing!

Existing circuits act slow as they rely on transcription and translation for signal progression, resulting in significant delays between input-sensing and toggling into the corresponding response state.

Not only Synthetic Biologists but nature itself controls important decisions—such as cell cycle progression, embryonal development or induced cell death—via bistable toggle switches. But nature knows how to act fast: rapid responses are not mediated by genetics but via post-translational protein modifications, such as phosphorylations.

Although phosphor-regulation has long been known, it was difficult to engineer and concert into designed behavior. Mishra *et al.* overcame this hurdle by developing (phosphorylation-required interaction and mediated effect (PRIME) that harnesses the modularity of natural phosphate regulators and allows us to build chimeric proteins that can be combined to ‘phosphor-in phosphor-out’ gates. One gate consists of two chimeric proteins that interact in a phosphate-dependent manner: once the upstream protein partner gets activated by a trigger, it binds to and activates or de-activates (phosphorylates or de-phosphorylates) its downstream partner. The downstream partner then acts as the activator within the next PRIME gate.

Using the PRIME gates, the authors build a network of logic gates resulting in a new-to-nature toggle network architecture that could be switched from one state to the other by two different chemical inputs, sorbitol and isopentenyl adenine—two chemicals for which receptors were readily available. As the first test read-out of the system, they used a green fluorescent protein that could be toggled between localization in the cytosol or the nucleus. Eventually, the authors showed to control a complex cellular function, yeast bud formation. State switching was thereby visualized in a microfluidic device allowing to expose cells to one of the two input signals and measure their switching pace.

The architecture of the toggle network was human-designed and had not been reported to be used by nature. As it resulted in a functional toggle behavior, the authors were interested to comprehensively test if nature could make the use of this architecture. They computationally identified several naturally occurring network motifs like the one they engineered. They experimentally verified that five of them encode a bistable behavior. In doing so, they contribute to demonstrating that synthetic biology can lead to a new understanding of natural system—one of the long-term goals of the field (4).

For their current PRIME design, the authors relied on naturally existing phosphorylation-induced interaction partners, just stitched together in new ways. The question remains if the approach can yield truly user-defined synthetic units. Scalable synthetic non-phosphorylated protein interaction units have been engineered before (5), as well as researchers have made progress in designing synthetic phosphorylation-dependent protein–protein interactions (6). Given the field of protein engineering is stronger than ever—thanks to directed evolution (7), artificial intelligence (8), as well as new protein design (9) and structure prediction tools (10)—there is reason to believe that we are only a few steps away from turning cells into real-time sensors for medicine and environmental surveillance.
